# Association of the atherogenic index of plasma with cardiovascular risk beyond the traditional risk factors: a nationwide population-based cohort study

**DOI:** 10.1186/s12933-022-01522-8

**Published:** 2022-05-22

**Authors:** Si Hyoung Kim, Yun Kyung Cho, Ye-Jee Kim, Chang Hee Jung, Woo Je Lee, Joong-Yeol Park, Ji Hye Huh, Jun Goo Kang, Seong Jin Lee, Sung-Hee Ihm

**Affiliations:** 1 Division of Endocrinology and Metabolism, Department of Internal Medicine, College of Medicine, Hallym University, Chuncheon, Republic of Korea; 2 Department of Clinical Epidemiology and Biostatistics, Asan Medical Center, University of Ulsan College of Medicine, Seoul, Republic of Korea; 3 Department of Internal Medicine, Asan Medical Center, University of Ulsan College of Medicine, Seoul, Republic of Korea; 4 Asan Diabetes Center, Asan Medical Center, Seoul, Republic of Korea

**Keywords:** Atherogenic index of plasma, Cardiovascular disease risk, Cardiovascular mortality, Mortality, Diabetes and endocrine research

## Abstract

**Background:**

The atherogenic index of plasma (AIP) is composed of triglycerides and high-density lipoprotein cholesterol and is a novel marker for assessing the risk of atherogenicity and cardiometabolic health. An association between AIP and greater frequency of major adverse cardiovascular events (MACEs) in patients with type 2 diabetes mellitus and high cardiovascular (CV) disease risk has been reported. However, only few studies have examined the correlation between AIP and CV risk in general populations. We thus aimed to evaluate the relationship between AIP and CV diseases using a large-scale population dataset from the Korean National Health Insurance Service-National Health Screening Cohort (NHIS-HEALS).

**Methods:**

A total of 514,866 participants were enrolled from the NHIS-HEALS and classified according to the AIP quartiles. We performed univariate and multivariate Cox proportional hazards regression analyses to determine the association between AIP and MACEs, CV events, and CV mortality.

**Results:**

During follow-up, we documented 12,133, 11,055, and 1942 cases of MACEs, CV events, and CV mortality, respectively. The multivariate-adjusted hazard ratios [HRs; 95% confidence interval (CI)] for MACEs gradually and significantly increased with the AIP quartiles [1.113 (1.054–1.175) in Q2, 1.175 (1.113–1.240) in Q3, and 1.278 (1.209–1.350) in Q4], following an adjustment for the conventional CV risk factors, including age, sex, body mass index, smoking, alcohol drinking, physical activities, household income, fasting glucose, systolic blood pressure, low-density lipoprotein cholesterol, and estimated glomerular filtration rate. In subgroup analyses, the association of AIP with MACEs and CV events was particularly outstanding in patients with diabetes.

**Conclusions:**

AIP was significantly associated with CV risks after adjusting for the traditional risk factors. Therefore, it may be used as an effective mass screening method to identify patients at a high risk of CV events.

**Supplementary information:**

The online version contains supplementary material available at 10.1186/s12933-022-01522-8.

## Background

Cardiovascular diseases (CVDs), namely ischemic heart disease (IHD) and stroke, are major causes of death and morbidity worldwide [1]. The prevalence of CVD has almost doubled in the last 20 years, from 271 million in 1990 to 523 million in 2019. During the similar period, the number of CVD deaths steadily increased from 12.1 million to 18.6 million [1]. In Korea, CVD is the second leading cause of mortality and accounted for 21.5% of all deaths in 2016, followed by malignancies (28.4%). However, as measured in disability-adjusted life years, the impact of circulatory system disorders was substantially larger than that of neoplasms, thereby indicating CVD imposes high economic healthcare burden [2, 3]. Researchers have identified multiple risk factors for CVD, such as type 2 diabetes mellitus (T2DM), obesity, hypertension, and dyslipidemia [4, 5]. Most recommendations advocate an individually tailored strategy based on established cardiovascular risk variables applied in risk stratification models [6–8]. However, clinicians regularly encounter patients with novel CVD events in clinical practice who have been misclassified by models based on conventional cardiovascular risk variables. This necessitates the development of efficient CVD risk estimates for the general population.

In recent years, researchers have recognized plasma lipid profile as a key risk factor for and predictor of CVD [9]. Dyslipidemia is defined as an increase in low-density lipoprotein cholesterol (LDL-C), total cholesterol, and triglyceride (TG) and a reduction in high-density lipoprotein cholesterol (HDL-C), which causes atherosclerosis [10]. Previously, LDL-C was assumed to be a major treatment target. Nonetheless, approximately half of the residual cardiovascular risks persisted following LDL-C decrease to the recommended levels, thus prompting researchers to discover novel CVD predictors [11]. In addition to individual serum cholesterol levels, including LDL-C, researchers have proposed the atherogenic index of plasma (AIP) [12], calculated using the formula log (TG/HDL-C), as a marker of plasma atherogenicity based on a positive association with the lipoprotein particle size, cholesterol esterification rates, and remnant lipoproteinemia [13, 14]. AIP not only accurately represents the link between protective and atherogenic lipoproteins but also acts as a powerful predictor of atherosclerosis and coronary heart disease [15]. A secondary analysis of the Action to Control Cardiovascular Risk in Diabetes (ACCORD) research revealed an association between AIP and greater frequency of major adverse cardiovascular events (MACEs) in patients with T2DM and high CVD risk [16].

In this background, this study aimed to assess the implication of AIP on MACEs, CV events, and CV mortality in participants who underwent a Korean national health screening test and to identify the particular subpopulation in which AIP is highly associated with CV risk and mortality.

## Methods

### Study population

**Fig. 1:**
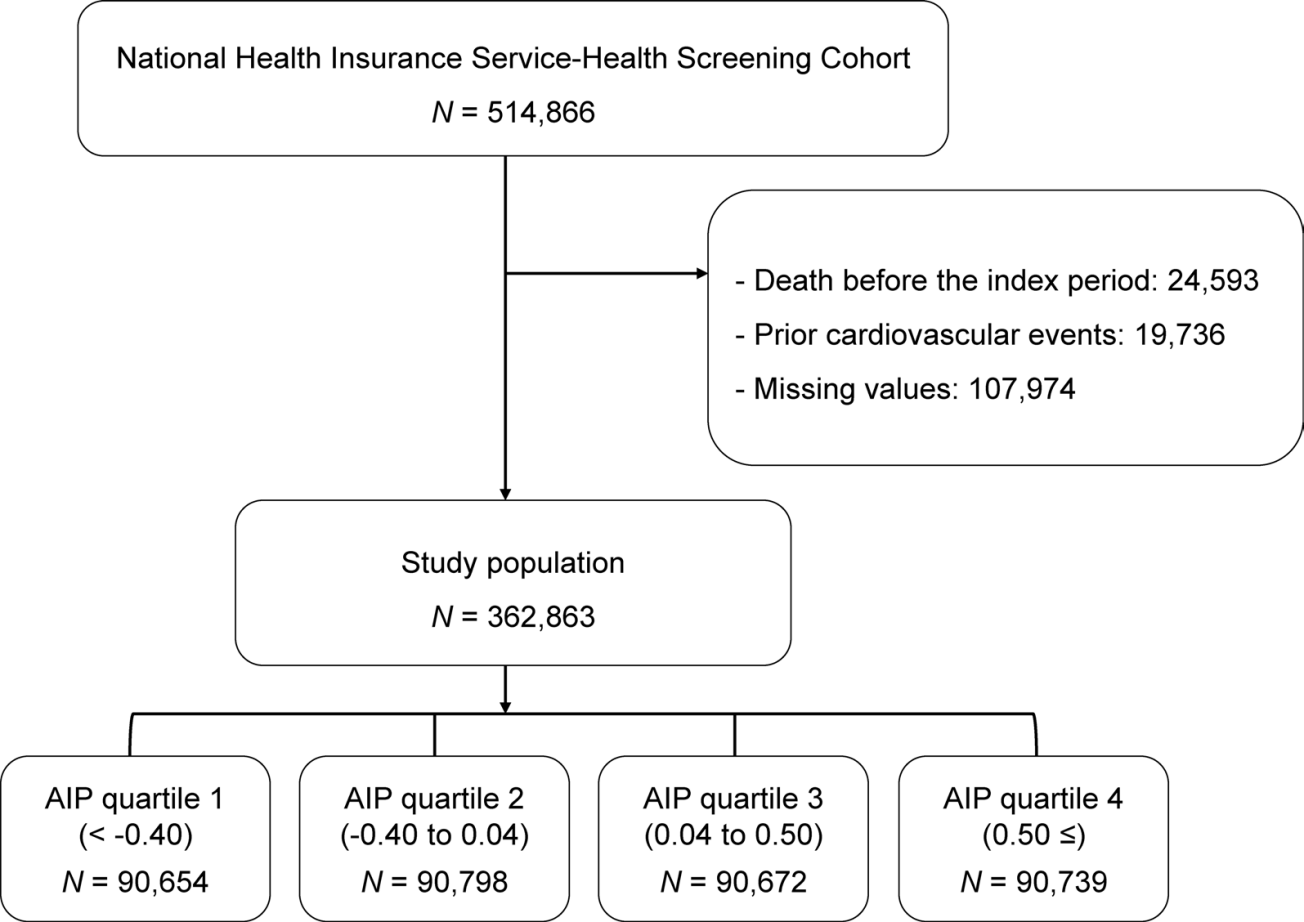
Study population enrollment.  *AIP* atherogenic index of plasma

We used data from the National Health Insurance-National Health Screening Cohort (NHIS-HEALS) from 2002 to 2015, provided by the Korean NHIS in 2017. The NHIS-HEALS comprised individuals who participated in health screening programs provided by the NHIS in the Republic of Korea. To construct the NHIS-HEALS database, a sample cohort was first selected from the 2002 and 2003 health screening participants aged between 40 and 79 years in 2002 and were followed up through 2015. To construct the NHIS-HEALS database, a sample cohort was first selected from the 2002 and 2003 health screening participants aged between 40 and 79 in 2002 and followed up through 2015. This cohort included 514,866 health screening participants who comprised a 10% simple random sample of all health screening participants in 2002 and 2003 [17]. The structure and function of the Korean NHIS-HEALS is explained in detail in the prior publication [17]. The index period was designated from January 10, 2009, to December 31, 2010, and was selected because of the inclusion of lipid profiles required to define metabolic health in the NHIS-HEALS since 2009 [17]. Among the 514,866 individuals in the NHIS-HEALS, we excluded patients who died or had a history of admission owing to a CV event before the conclusion of the index period. The research cohort included 362,863 patients. Figure 1 depicts the study participants and design. The Hallym Institutional Review Board authorized this research (reference number HALLYM 2021-05-007). Anonymous and de-identified information was used for analysis; therefore, informed consent was not obtained.

### Calculation of AIP

The AIP is a logarithmically converted ratio of TG to HDL-C in molar concentration (millimole per liter), which can be analytically calculated from log (TG/HDL-C) [18]. Subsequently, we classified the study population into four groups according to the AIP quartiles (Q1, − 0.10; Q2, − 0.10, 0.08; Q3, 0.08, 0.26; and Q4, 0.26).

### Definition of outcomes

The primary endpoint included MACEs, which were a composite of nonfatal myocardial infarction (MI), nonfatal stroke, and CV mortality. CV events were defined as MI and stroke (ischemic or hemorrhagic) admissions between January 1, 2011, and December 31, 2015. Occurrence was identified using the hospital discharge data. We included participants with MI or stroke as one of the 10th version of the International Classification of Diseases (ICD-10) codes for a primary or secondary illness. Information on mortality and the cause of death was available for all participants. The Korean National Statistical Office publishes the Korean Standard Classification of Diseases and Causes of Death, based on ICD-10. Diseases or disorders that directly caused death were considered the cause of death. CV mortalities were classified as deaths caused by circulatory system diseases (I00–99).

### Definitions of type 2 diabetes, hypertension, and dyslipidemia

T2DM was defined as prescription of antidiabetic drugs and reporting of ICD-10 codes E11 (non-insulin-dependent diabetes mellitus), E12 (malnutrition-related diabetes mellitus), E13 (other specified diabetes mellitus), and E14 (unspecified diabetes mellitus) as the primary or secondary diagnosis. During the study, pharmacies in Korea supplied the following eight types of diabetes medications: sulfonylureas, biguanides, glucosidase inhibitors, thiazolidinediones, meglitinide, glucagon-like peptide-1 receptor agonists, dipeptidyl peptidase-4 inhibitors, and insulin.

Participants with ICD-10 codes I10 (essential [primary] hypertension), I11 (hypertensive heart disease), I12 (hypertensive chronic kidney disease), I13 (hypertensive heart and chronic kidney disease), and I15 (secondary hypertension) as the primary or secondary diagnosis were classified as having hypertension. During the study, the aforementioned pharmacies issued the following five types of antihypertensive medications: angiotensin receptor blockers, angiotensin-converting enzyme inhibitors, beta blockers, calcium channel blockers, and diuretics. Dyslipidemia was defined as use of lipid-lowering drugs and the reporting of the ICD-10 code E78 (disorders of lipoprotein metabolism and other lipidemias) as the primary or secondary diagnosis. Statins, ezetimibe, and fibrates were among the lipid-lowering drugs dispensed by pharmacies throughout the study.

### Covariates

Covariates from the baseline health examination included smoking habits (non-smoker, ex-smoker, or current smoker), drinking habits (none, mild, moderate, or heavy drinking), physical activity (0, 1–2, 3–4, or 5 times per week), LDL-C, and estimated glomerular filtration rate (eGFR). Heavy drinkers were defined as those who consumed seven or more drinks on an occasion and drank more than 5 days per week. Contrarily, mild and moderate drinkers consumed seven drinks on any one day and drank 1–2 or 3–4 days per week, respectively.

### Statistical analysis

We estimated the hazard ratio (HR) and 95% confidence interval (CI) of MACEs, CV events, and CV mortality using Cox proportional hazards models. Age, sex, body mass index (BMI), smoking, alcohol consumption, physical activities, household income, fasting glucose, systolic blood pressure, LDL-cholesterol level, and eGFR were factored into multivariate models. We plotted a restricted cubic spline of log hazard for MACEs, CV events, and CV mortality as continuous variables on a logarithmic scale to examine their association with AIP. The SAS Enterprise Guide software was used for all statistical analyses (version 7.1, SAS Institute, Inc., Cary, NC, USA).

## Results

### Baseline characteristics of the entire cohort

Table 1 summarizes the baseline clinical and biochemical characteristics of the participants according to their AIP quartile. AIP was associated with atherosclerotic CV disease risk factors and metabolic syndrome components, such as BMI, current smoking, alcohol consumption, systolic and diastolic blood pressure, fasting glucose, total cholesterol, LDL-C, and high prevalence of hypertension, diabetes, and dyslipidemia (all p < 0.001). Physical activity was lower in those with increased AIP (all p < 0.001). AIP demonstrated an inverse relationship with eGFR (all p < 0.001).

**Table 1:** Characteristics of the study participants according to the AIP quartiles

	Q1 (< – 0.40)	Q2 (– 0.40 to 0.04)	Q3 (0.04 to 0.50)	Q4 (0.50 ≤)	p-value
N	90,654	90,798	90,672	90,739	
Sex (% men)	41.7	49.5	56.6	67.0	< 0.0001
Age (years)	58.6 ± 8.7	59.4 ± 8.8	59.4 ± 8.8	58.5 ± 8.5	< 0.0001
BMI (kg/m^2^)	23.0 ± 2.8	23.9 ± 2.9	24.5 ± 2.8	25.1 ± 2.8	< 0.0001
WC (cm)	78.5 ± 8.1	81.5 ± 8.0	83.7 ± 7.8	85.9 ± 7.5	< 0.0001
Systolic BP (mmHg)	123.2 ± 15.2	125.9 ± 15.2	127.7 ± 15.0	129.9 ± 14.9	< 0.0001
Diastolic BP (mmHg)	76.1 ± 9.9	77.9 ± 9.9	79.1 ± 9.8	80.8 ± 9.9	< 0.0001
Smoking (%)					< 0.0001
Current smoker	10.3	14.5	18.6	26.3	
Ex-smoker	14.4	17.1	19.3	22.1	
Non-smoker	72.3	65.3	59.1	48.9	
Drinking (%)					< 0.0001
None	61.8	59.8	56.3	49.2	
Mild	17.4	16.9	17.0	16.8	
Moderate	4.2	4.3	4.3	4.6	
Heavy	14.0	16.4	20.0	27.5	
Physical activity (%)					< 0.0001
None	26.1	27.8	29.1	30.5	
1–2 times/week	19.8	21.7	23.0	25.4	
3–4 times/week	21.6	21.1	20.9	20.4	
≥ 5 times/week	30.4	27.2	25.0	22.1	
Hypertension (%)	21.1	27.1	31.4	35.6	
Diabetes (%)	5.1	7.4	9.6	13.1	
Dyslipidemia (%)	13.3	17.2	19.9	24.7	
FPG (mg/dL)	97.3 ± 20.0	100.5 ± 22.5	103.4 ± 25.5	109.4 ± 32.9	< 0.0001
TG (mg/dL)	71.1 ± 31.9	106.6 ± 31.0	147.1 ± 31.3	262.9 ± 115.1	< 0.0001
HDL-C (mg/dL)	68.1 ± 43.8	55.4 ± 16.2	49.3 ± 9.4	42.3 ± 8.7	< 0.0001
LDL-C (mg/dL)	117.9 ± 37.0	123.9 ± 36.3	126.0 ± 37.2	116.9 ± 45.1	< 0.0001
TC (mg/dL)	197.1 ± 35.3	200.7 ± 36.8	204.8 ± 37.5	209.7 ± 39.8	< 0.0001
eGFR (mL/min/1.73 m^2^)	82.2 ± 18.7	80.2 ± 18.8	78.9 ± 19.2	77.9 ± 19.9	< 0.0001

Results reported as means ± standard deviation (SD) or percentage, unless otherwise indicated

BMI: body mass index; BP: blood pressure; eGFR: estimated glomerular filtration rate; FPG: fasting plasma glucose; HDL-C: high-density lipoprotein cholesterol; LDL-C: low-density lipoprotein cholesterol; TC: total cholesterol; TG: triglyceride; WC: waist circumference

### MACEs, CV events, and CV mortality according to the AIP quartile

**Fig. 2:**
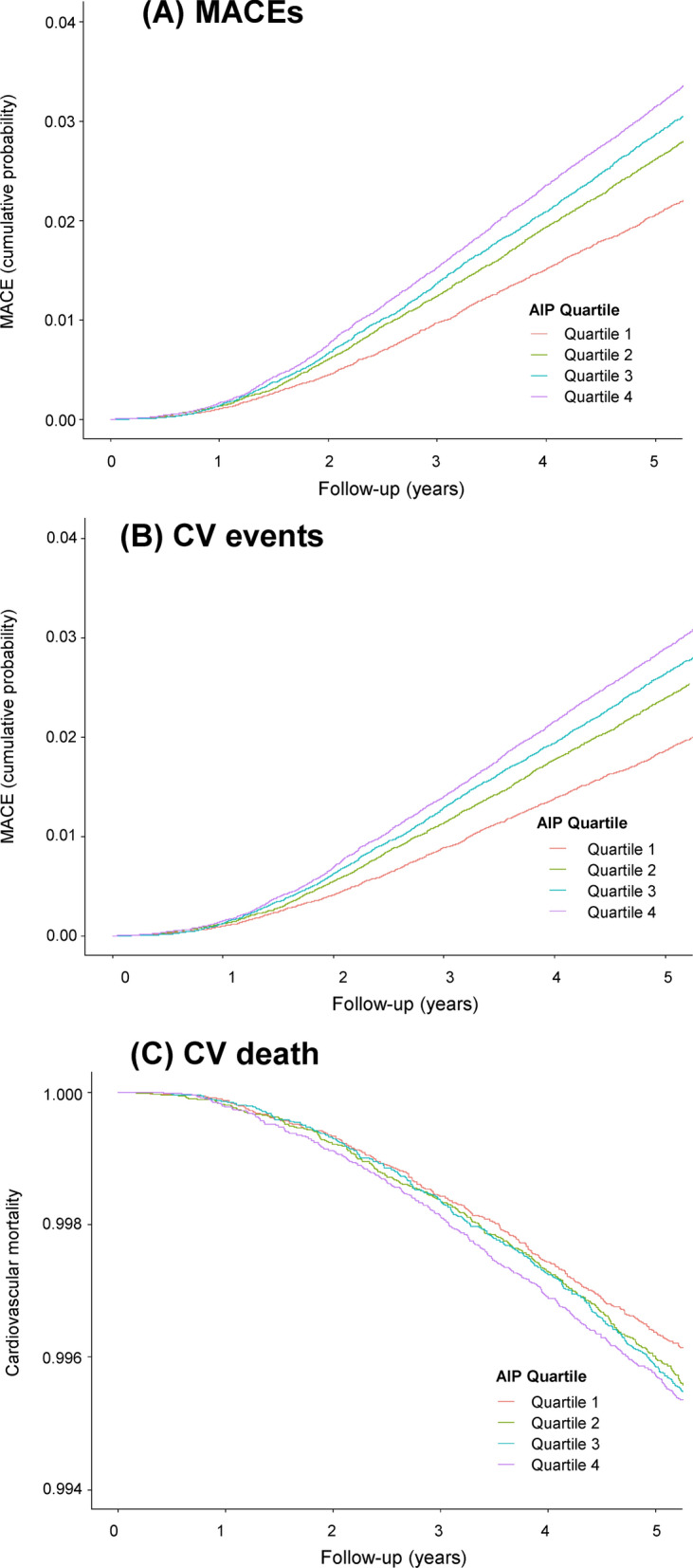
Kaplan–Meier curves for cardiovascular outcomes and mortality according to the AIP quartiles. **A** MACEs, **B** CV events, and **C** CV mortality. AIP, atherogenic index of plasma; MACE, major adverse cardiovascular event; CV, cardiovascular

Of the 362,863 individuals, we identified 12,133, 11,055, and 1942 cases of MACEs, CV events, and CV mortality, respectively. Figure 2 depicts the Kaplan–Meier curves for the cumulative incidence of MACEs, CV events, and CV mortality. The highest AIP quartile was associated with the greatest probability of developing MACEs, CV events, and CV mortality. In MACEs and CV events, the probabilities decreased sequentially for lower quartiles (both log rank p < 0.001).

**Fig. 3:**
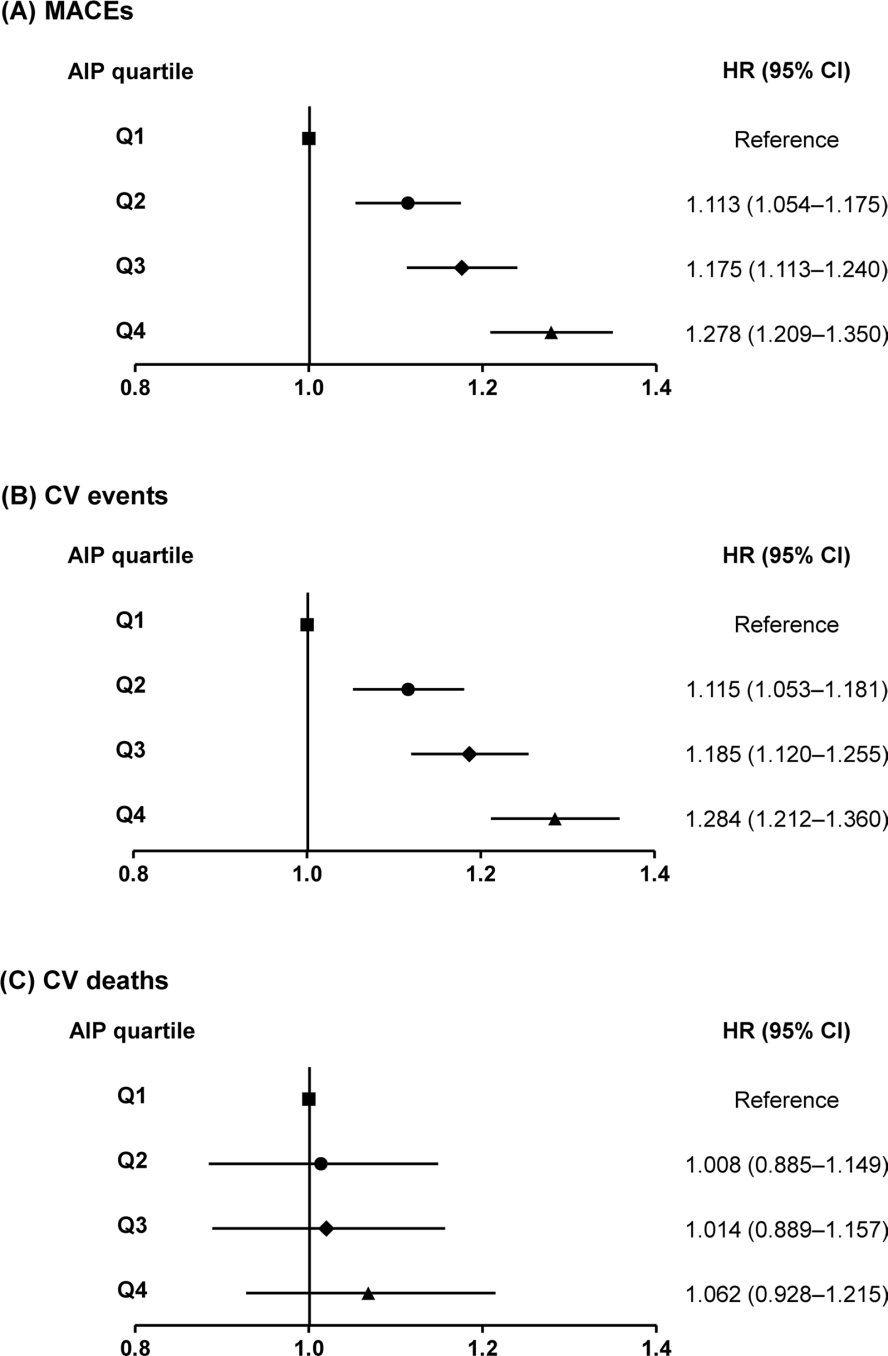
Summarized figure of HRs for (**A**) MACEs, (**B**) CV events, and (**C**) CV mortality. HR, hazard ratio; CI, confidence interval; MACE, major adverse cardiovascular event; CV, cardiovascular, AIP, atherogenic index of plasma, Q, quartile

During the follow-up, 3,578 patients with the highest AIP (Q4) developed MACEs (overall incidence of 3.96%), whereas only 2342 patients with the lowest AIP (Q1) reported similar outcomes (overall incidence of 2.58%) (Table 2). The age- and sex-adjusted HRs for MACEs increased for the 2nd (1.174, 95% CI 1.112–1.240), 3rd (1.296, 95% CI 1.229–1.367), and 4th (1.503, 95% CI 1.427–1.584) AIP quartiles, compared with that for the 1st quartile (Table 2). Moreover, the age- and sex-adjusted HRs for CV events increased as follows: 1.178 (95% CI 1.113–1.248), 1.311 (95% CI 1.240–1.386), and 1.515 (95% CI 1.434–1.600) for the 2nd, 3rd, and 4th quartiles, respectively, compared with that for the 1st quartile (Table 2). CV mortality significantly increased in participants with the highest AIP quartile, i.e., 1.222 (95% CI 1.075–1.390) for the 4th quartile compared with that for the 1st quartile (Table 2). The multivariate-adjusted model for age, sex, BMI, smoking, alcohol drinking, physical activities, household income, fasting glucose, systolic blood pressure, LDL-cholesterol level, and eGFR revealed a significant and progressive increase in the risk of MACEs (HR [95% CI]: 1.113 [1.054–1.175] for the 2nd, 1.175 [1.113–1.240] for 3rd, and 1.278 [1.209–1.350] for 4th AIP quartiles) and CV events (HR [95% CI]: 1.115 [1.053–1.181] for the 2nd, 1.185 [1.120–1.255] for 3rd, 1.284 [1.212–1.360] for 4th AIP quartiles) with increasing AIP quartiles (Table 2). However, the association between CV mortality and the AIP quartile was insignificant following multivariable adjustment (Table 2). Figure 3 summarizes the multivariable-adjusted HRs for MACEs, CV events, and CV mortality according to the AIP quartiles. Moreover, the restricted cubic splines of HRs for MACEs, CV events, and CV mortality demonstrated that the levels of AIP associated with an increased CV risk were 0.77 and 0.78 for MACEs and CV events, respectively (Additional file 1: Fig. S1).

**Table 2:** Hazard ratios for (A) MACEs, (B) CV events, and (C) CV mortality according to AIP quartiles

	Event N (%)	Model 1	Model 2	Model 3
(A) MACEs				
Q1	2342/90,654 (2.58)	Reference	Reference	Reference
Q2	2951/90,798 (3.25)	1.259 (1.192–1.329)	1.174 (1.112–1.240)	1.113 (1.054–1.175)
Q3	3262/90,672 (3.60)	1.395 (1.323–1.471)	1.296 (1.229–1.367)	1.175 (1.113–1.240)
Q4	3578/90,739 (3.96)	1.533 (1.455–1.615)	1.503 (1.427–1.584)	1.278 (1.209–1.350)
(B) CV events				
Q1	2123/90,654 (2.34)	Reference	Reference	Reference
Q2	2677/90,798 (2.36)	1.260 (1.190–1.334)	1.178 (1.113–1.248)	1.115 (1.053–1.181)
Q3	2984/90,672 (2.96)	1.408 (1.332–1.489)	1.311 (1.240–1.386)	1.185 (1.120–1.255)
Q4	3271/90,739 (3.28)	1.546 (1.464–1.633)	1.515 (1.434–1.600)	1.284 (1.212–1.360)
(C) CV mortality				
Q1	428/90,654 (0.47)	Reference	Reference	Reference
Q2	491/90,798 (0.48)	1.142 (1.003–1.299)	1.044 (0.917–1.188)	1.008 (0.885–1.149)
Q3	509/90,672 (0.56)	1.184 (1.042–1.347)	1.094 (0.962–1.244)	1.014 (0.889–1.157)
Q4	514/90,739 (0.56)	1.196 (1.052–1.360)	1.222 (1.075–1.390)	1.062 (0.928–1.215)

AIP: Atherogenic index of plasma; MACE: major adverse cardiovascular event; CV: cardiovascular; Q: quartile

Model 1, unadjusted; Model 2, adjusted for age and sex; and Model 3, adjusted for baseline age, sex, body mass index, smoking, alcohol drinking, physical activities, household income, fasting glucose, systolic blood pressure, low-density lipoprotein cholesterol, and estimated glomerular filtration rate levels.

### Subgroup analyses

Associations between AIP and MACEs, CV events, and CV mortality was generally consistent across the subgroups according to the clinical variables, including known individual CV risk factors, following multivariate adjustment (Additional file 1: Fig. S2). With regard to underlying diseases, high AIP was significantly associated with MACEs, CV events, and even CV mortality in patients with diabetes (HR [95% CI]: 1.279 [1.174 − 1.395], 1.279 [1.168 − 1.400], and 1.337 [1.083 − 1.650] for MACEs, CV events, and CV mortality, respectively); however, AIP was still useful in those without diabetes (HR [95% CI]: 1.118 [1.072 − 1.166] and 1.127 [1.079 − 1.177] for MACEs and CV events, respectively). In the subgroup with LDL-C ≥ 70 mg/dL, participants with high AIP had a higher risk of MACEs and CV events (HR [95% CI]: 1.163 [1.119 − 1.209] and 1.169 [1.122 − 1.217], respectively). However, in individuals with LDL-C < 70 mg/dL, the association between AIP and CV risk was not significant (HR [95% CI]) for high AIP vs. low AIP (0.993 [0.865−1.138]) and MACEs vs. CV events (1.012 [0.874 − 1.171]).

## Discussion

Using a large-scale nationwide cohort dataset, we explored the relationship between AIP and CV risk. High AIP was related to a considerably greater risk of future MACEs, despite controlling for potential confounders, such as CV risk factors. Our results showed that the HRs for both MACEs and CV events in the highest quartiles (or the lowest quartiles for HDL-C) were even higher when AIP was applied than that when TG or HDL-C were applied alone (Table 2, Additional file 1: TableS1, 2). Although we encourage researchers to cautiously review findings of any observational study, this large countrywide observational study included more than 360,000 individuals, demonstrating that high AIP was significantly associated with future CV events.

AIP is defined by the ratio of TG and HDL-C [19], and TG is directly associated with serum LDL-C levels [20]. Therefore, the underlying explanation for the relationship between AIP and CVD incidence is most likely related to its correlation with the lipoprotein particle size [21]. AIP is a substitute for minute dense LDL particles and is inversely related to the LDL-C particle diameter. Therefore, an increase in AIP indicates an upsurge in the fraction of particles prone to oxidation and, in turn, the production of foamy cells. Contrarily, this tendency leads to an increase in the LDL-C and oxidized apoprotein B combination, known for its strong atherogenicity. Endothelial dysfunction is directly correlated to AIP because it promotes lipid peroxidation and activates oxygen radicals and overexpression of adhesion molecules [22]. HDL-C is an additional feature of AIP, which transports peripheral cholesterol to the liver and includes antioxidant enzymes, such as paraoxonase [23]. These theoretical considerations have been practically verified by the direct association between AIP and carotid artery intima-media thickness [24], arterial stiffness [25], and coronary artery calcification [26].

AIP could be considered an independent predictor of future CV events using a large, nationwide cohort dataset, thereby implying it could reflect the overall state of atherosclerosis [16]. Participants in the highest AIP quartile demonstrated a 28% greater risk of MACEs than those in the lowest quartile. Moreover, the risk was primarily generated from CV events, considering an insignificant increase in CV mortality according to AIP. Our subgroup analysis revealed that the association between AIP and MACEs or CV events remained significant following multivariable adjustment for participants with diabetes, with a HR of 1.285 (1.179−1.401) and 1.284 (1.173−1.406) for MACEs and CV events, respectively. This finding was consistent with those of the ACCORD project, which randomized 10,251 participants with long-term diabetes [16]. After controlling for the traditional risk variables, the correlation between AIP and CV mortality was also significant in patients with diabetes (HR [95% CI]: 1.348 [1.092−1.664]), while an increase in HRs with higher AIP was not significant in the non-diabetic population (HR [95% CI]: 0.980 [0.883−1.088]). These findings suggest the clinical usefulness of AIP for estimating future CV risk particularly in patients with diabetes.

Unlike the previous ACCORD study, we were able to investigate the association between AIP and CV outcomes in a non-diabetic population. This is because our study included the general population with national health assessments. In the subgroup analyses in our study, the significant association between AIP and MACEs persisted for participants without diabetes, albeit at a reduced HR (HR [95% CI]: 1.279 [1.174−1.385] with diabetes vs. 1.118 [1.072−1.166] without diabetes), thus extending the clinical utility of AIP for CV risk estimation. Kim et al. demonstrated that increased AIP levels were positively and independently correlated with IHD in 17,944 people without diabetes [27]. Sadeghi et al. [19] further mentioned that AIP was an independent predictor of CV events in 6,323 healthy persons aged > 35 years from 2001 to 2016. Thus, in conjunction with prior results, we proposed that AIP could be an effective surrogate marker for future CV events in the general population, including participants with and without diabetes. However, after controlling for the traditional risk variables, the correlation between AIP and CV mortality became insignificant, despite an increase in HRs with higher AIP (HR [95% CI]: 0.980 [0.883 − 1.088]). The duration of the study could be the primary reason for this finding. Our participants were followed up from the index period (from 2009 to 2010) to 2015; thus, this relatively short follow-up may be insufficient to comprehensively examine the interactions, particularly for CV mortality. Therefore, lengthier follow-up could yield different results. Furthermore, our subgroup analyses revealed that in individuals with LDL-C < 70 mg/dL, the association between AIP and CV risk was not significant (HR [95% CI]) for high AIP vs. low AIP (0.993 [0.865−1.138]) and for MACEs vs. CV events (1.012 [0.874−1.171]). The small number of participants with LDL-C < 70 mg/dL and relatively low level of AIP (0.01 ± 0.58 in LDL-C < 70 group vs. 1.20 ± 0.70 in LDL-C > 70 group) in this group could affect the discriminative function of AIP in this population. However, further studies with larger number of participants are warranted to explain this finding.

This study has some limitations. First, we only included Korean individuals; thus, our findings may not be generalizable to other ethnic groups. Second, fewer events and short follow-up periods may have underpowered the study to appropriately assess the interactions. Third, our claims data-based definition of CV events may not be completely trustworthy. To increase the accuracy, we defined the outcomes by integrating the diagnostic and prescription histories. Fourth, a large proportion of participants were excluded from our analysis, and the exclusion was mainly owing to missing values. Among participants who were excluded, the proportion of males, older people, and low-income participants was higher, which can affect the study results (Additional file 1: Table S3). However, after adjusting the confounding factors, including sex, age, and household income, the association between AIP and MACEs remained significant. Further, despite controlling for CV risk factors, there may have been other confounding factors. Moreover, certain undisclosed aspects may have influenced our results, even after correcting the analyses for most accessible demographic and clinical factors. Finally, a significant association does not always establish a claim of prediction [28, 29], indicating that our results do not guarantee the predictive validity of AIP in individual levels. However, our results still bear clinically important information, in that we have demonstrated the robust association of AIP, a simple biomarker using two important lipid profiles, with the risk of future CV events.

The strengths of our study include the use of a large-scale, countrywide database and demonstrating the association of AIP with future MACEs through an adjusted analysis with multiple confounding variables and subgroup analyses. Our findings demonstrated that AIP may be used to estimate future CV risk in clinical settings at a reasonable cost. Researchers have attempted to identify persons at a higher risk of CVD and to prevent the development of overt CVD. This resulted in the development of various risk calculators for calculating CVD risk in the general population. Important risk factors include hypertension, smoking, diabetes, hyperlipidemia, obesity, and a family history of CVD. By contrast, accurate data collection is time-consuming and frequently necessitates the involvement of a third party while administering a questionnaire. Contrarily, AIP can be computed relatively quickly with a one-time fasting blood test; when combined with our findings, AIP may be considered a novel MACE bioindicator to assess future CV event risk.

## Conclusions

In our study, greater AIP was related to a higher risk of MACEs in this large nationwide population-based cohort. In particular, participants with higher AIP were at higher risk of future CV events if they have diabetes. This index may be utilized as an effective mass screening method to identify patients at a high risk of CV events.

## Supplementary Information

**Additional file 1: Table S1.**Hazardratios for (A) MACEs, (B) CV events, and (C) CVmortality according to TG quartiles.**Table S2.**Hazardratios for (A) MACEs, (B) CV events, and (C) CV mortality according to HDLquartiles (reversed). **Table S3.**Characteristics of study participants who were included in the analyses versusthose who were excluded from the analyses.**Figure S1.** Cubic spline curves of hazard ratios forMACEs, CV events, and CV mortality according to AIP levels.** Figure S2.** Subgroupanalyses for the risk of (A) MACEs, (B) CV events, and (C) CV mortality. 

## Data Availability

The datasets generated and analyzed during the current study are not publicly available due to the rules of the Korean National Health Insurance System.

## Abbreviations

ICD-10: 10Th version of the International Classification of Diseases
ACCORD: Action to Control Cardiovascular Risk in Diabetes
AIP: Atherogenic index of plasma
CV: Cardiovascular
CI: Confidence interval
EGFR: Estimated glomerular filtration rate
HR: Hazard ratio
HDL-C: High-density lipoprotein cholesterol
IHD: Ischemic heart disease
LDL-C: Low-density lipoprotein cholesterol
MACE: Major adverse cardiovascular event
MI: Myocardial infarction
NHIS-HEALS: National Health Insurance Service-National Health Screening Cohort
TG: Triglyceride
T2DM: Type 2 diabetes mellitus
