# Supplementary material for: Association of the atherogenic index of plasma with cardiovascular risk beyond the traditional risk factors: a nationwide population-based cohort study
Source: Cardiovasc Diabetol. 2022 May 22;21:81. doi: 10.1186/s12933-022-01522-8 (PMC9124430; doi:10.1186/s12933-022-01522-8)

**Additional Materials to:**

Si Hyoung Kim et.al. “Association of the atherogenic index of plasma with cardiovascular risk beyond the traditional risk factors: A nationwide population-based cohort study”

**Contents**

**Additional tables**

**Table S1.** Hazard ratios for (A) MACEs, (B) CV events, and (C) CV mortality according to TG quartiles

**Table S2.** Hazard ratios for (A) MACEs, (B) CV events, and (C) CV mortality according to HDL quartiles (reversed).

**Table S3.** Characteristics of study participants who were included in the analyses versus those who were excluded from the analyses.

**Additional figures**

**Figure S1.** Cubic spline curves of hazard ratios for MACEs, CV events, and CV mortality according to AIP levels.

**Figure S2.** Subgroup analyses for the risk of (A) MACEs, (B) CV events, and (C) CV mortality

**Table S1.** Hazard ratios for (A) MACEs, (B) CV events, and (C) CV mortality according to TG quartiles

|  |  | **Event N (%)** | **Model 1** | **Model 2** | **Model 3** |
| --- | --- | --- | --- | --- | --- |
| **(A) MACEs** | **Q1** | 2404/88,554 (2.71) | Reference | Reference | Reference |
|  | **Q2** | 2918/90,954 (2.70) | 1.181 (1.119–1.247) | 1.126 (1.067–1.189) | 1.059 (1.003–1.118) |
|  | **Q3** | 3292/91,845 (3.19) | 1.320 (1.252–1.391) | 1.250 (1.186–1.318) | 1.126 (1.067–1.188) |
|  | **Q4** | 3519/91,510 (3.60) | 1.420 (1.349–1.496) | 1.439 (1.367–1.516) | 1.213 (1.149–1.281) |
| **(B) CV events** | **Q1** | 2167/88,554 (2.45) | Reference | Reference | Reference |
|  | **Q2** | 2653/90,954 (2.46) | 1.191 (1.126–1.261) | 1.137 (1.074–1.204) | 1.068 (1.008–1.131) |
|  | **Q3** | 3007/91,845 (2.91) | 1.337 (1.266–1.413) | 1.267 (1.199–1.339) | 1.138 (1.076–1.204) |
|  | **Q4** | 3228/91,510 (3.28) | 1.445 (1.369–1.526) | 1.459 (1.382–1.541) | 1.227 (1.159–1.299) |
| **(C) CV mortality** | **Q1** | 442/88,554 (0.50) | Reference | Reference | Reference |
|  | **Q2** | 477/90,954 (0.49) | 1.047 (0.920–1.191) | 0.990 (0.870–1.127) | 0.942 (0.827–1.074) |
|  | **Q3** | 534/91,845 (0.52) | 1.158 (1.021–1.314) | 1.104 (0.973–1.252) | 1.015 (0.892–1.154) |
|  | **Q4** | 489/91,510 (0.59) | 1.066 (0.938–1.213) | 1.152 (1.013–1.310) | 0.986 (0.861–1.129) |

Abbreviations: MACE, major adverse cardiovascular event; CV, cardiovascular; Q, quartile

Model 1, unadjusted; Model 2, adjusted for age and sex; and Model 3, adjusted for baseline age, sex, body mass index, smoking, alcohol drinking, physical activities, household income, fasting glucose, systolic blood pressure, low-density lipoprotein cholesterol, and estimated glomerular filtration rate levels.

**Table S2.** Hazard ratios for (A) MACEs, (B) CV events, and (C) CV mortality according to HDL quartiles (reversed).

|  |  | **Event N (%)** | **Model 1** | **Model 2** | **Model 3** |
| --- | --- | --- | --- | --- | --- |
| **(A) MACEs** | **Q4** | 2895/99,227 (2.92) | Reference | Reference | Reference |
|  | **Q3** | 2752/90,675 (2.93) | 1.039 (0.986–1.095) | 1.030 (0.977–1.085) | 1.010 (0.958–1.064) |
|  | **Q2** | 3220/92,181 (3.04) | 1.197 (1.139–1.259) | 1.148 (1.091–1.207) | 1.094 (1.039–1.151) |
|  | **Q1** | 3266/80,780 (3.50) | 1.390 (1.322–1.462) | 1.288 (1.225–1.355) | 1.181 (1.121–1.243) |
| **(B) CV events** | **Q4** | 2635/99,227 (2.66) | Reference | Reference | Reference |
|  | **Q3** | 2527/90,675 (2.67) | 1.048 (0.993–1.107) | 1.038 (0.983–1.097) | 1.016 (0.962–1.074) |
|  | **Q2** | 2944/92,181 (2.80) | 1.203 (1.203–1.141) | 1.155 (1.096–1.218) | 1.099 (1.042–1.160) |
|  | **Q1** | 2949/80,780 (8.19) | 1.379 (1.309–1.454) | 1.284 (1.218–1.354) | 1.177 (1.115–1.243) |
| **(C) CV mortality** | **Q4** | 514/99,227 (0.52) | Reference | Reference | Reference |
|  | **Q3** | 421/90,675 (0.51) | 0.894 (0.786–1.017) | 0.902 (0.793–1.026) | 0.905 (0.795–1.030) |
|  | **Q2** | 480/92,181 (0.48) | 1.002 (0.885–1.135) | 0.962 (0.849–1.090) | 0.935 (0.825–1.061) |
|  | **Q1** | 527/80,780 (0.51) | 1.256 (1.112–1.418) | 1.145 (1.013–1.294) | 1.069 (0.943–1.212) |

Abbreviations: MACE, major adverse cardiovascular event; CV, cardiovascular; Q, quartile

Model 1, unadjusted; Model 2, adjusted for age and sex; and Model 3, adjusted for baseline age, sex, body mass index, smoking, alcohol drinking, physical activities, household income, fasting glucose, systolic blood pressure, low-density lipoprotein cholesterol, and estimated glomerular filtration rate levels.

**Table S3.** Characteristics of study participants who were included in the analyses versus those who were excluded from the analyses.

|  |  | **Excluded** | | **Included** | |  |
| --- | --- | --- | --- | --- | --- | --- |
|  |  | **N** | **%** | **N** | **%** | **P** |
|  |  | 152,003 | 100.0% | 362,863 | 100.0% |  |
| **Sex** | **Male** | 84,264 | 55.4% | 194,861 | 53.7% | <.001 |
|  | **Female** | 67,739 | 44.6% | 168,002 | 46.3% |  |
| **Age at HEALS entry** | **mean SD** | 55.44 | 11.01 | 51.47 | 8.74 | <.001 |
| **Age group** | **40–49** | 57,610 | 37.9% | 179,371 | 49.4% | <.001 |
|  | **50–59** | 36,831 | 24.2% | 108,201 | 29.8% |  |
|  | **60–69** | 37,292 | 24.5% | 63,864 | 17.6% |  |
|  | **70–79** | 20,270 | 13.3% | 11,427 | 3.1% |  |
| **Household income** | **Medicaids** | 378 | 0.2% | 378 | 0.2% | <.001 |
|  | **Quintile: 1** | 28,195 | 18.5% | 53,275 | 14.7% |  |
|  | **Quintile: 2** | 23,756 | 15.6% | 50,313 | 13.9% |  |
|  | **Quintile: 3** | 24,339 | 16.0% | 57,362 | 15.8% |  |
|  | **Quintile: 4** | 29,185 | 19.2% | 76,592 | 21.1% |  |
|  | **Quintile: 5** | 46,150 | 30.4% | 125,184 | 34.5% |  |

HEALS, health screening cohort; SD, standard deviation

**Figure S1.** Cubic spline curves of hazard ratios for MACEs, CV events, and CV mortality according to AIP levels.

The levels of AIP which increase the risk level of each outcome are indicated in red, and the median and the interquartile ranges of AIP are indicated in black.

AIP, atherogenic index of plasma; MACE, major adverse cardiovascular event; CV, cardiovascular


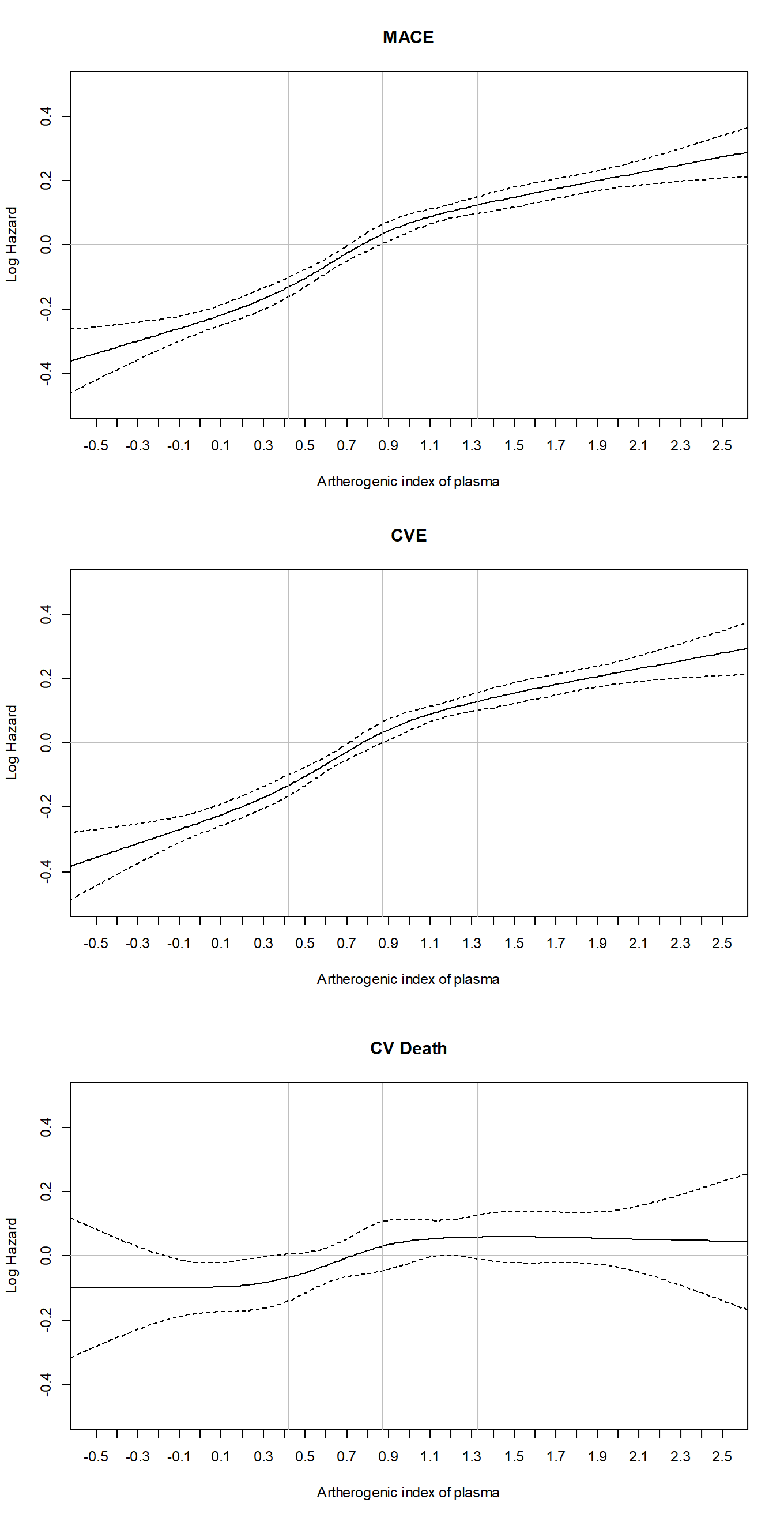
0

**Figure S2.** Subgroup analyses for the risk of (A) MACEs, (B) CV events, and (C) CV mortality

The high (upper half) and low (lower half) AIP groups have been compared after adjusting for age, sex, BMI, smoking, alcohol drinking, physical activities, fasting glucose, systolic BP, LDL-cholesterol, and eGFR levels. The covariates are excluded from the adjustment in the corresponding subgroup analyses.

AIP, atherogenic index of plasma; MACE, major adverse cardiovascular event; CV, cardiovascular; BMI, body mass index; eGFR, estimated glomerular filtration rate; BP, blood pressure; and LDL-cholesterol, low-density lipoprotein cholesterol.


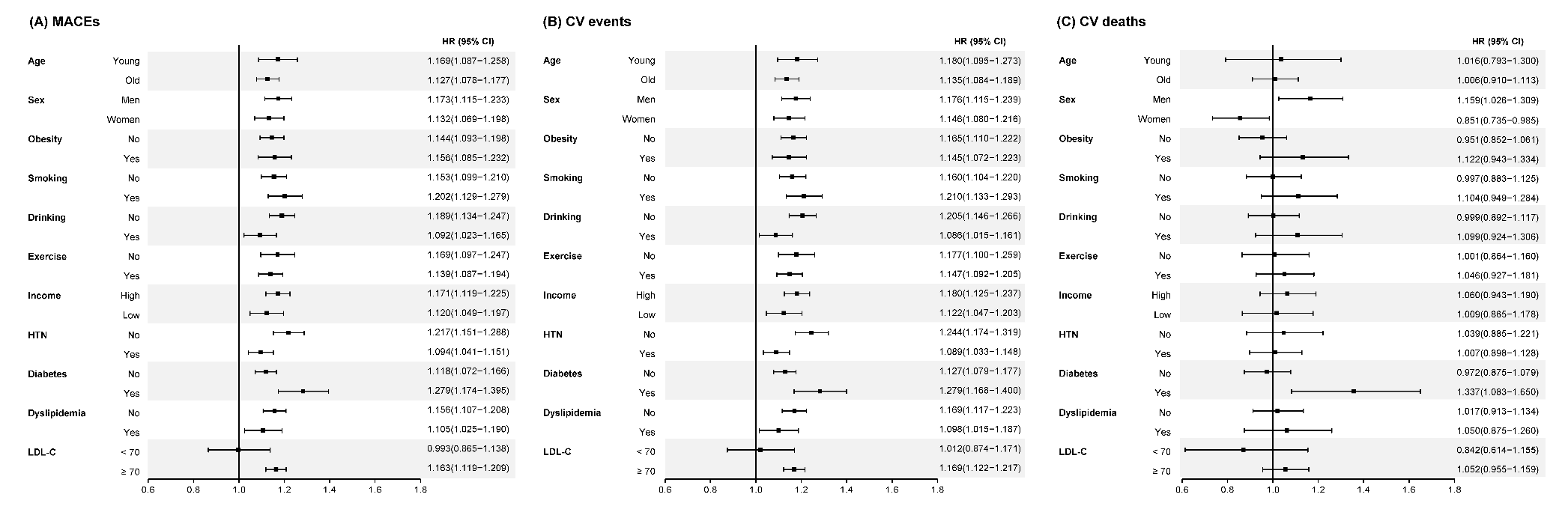

Supplement: Supplementary file 1 — Additional file 1: Table S1.Hazardratios for (A) MACEs, (B) CV events, and (C) CVmortality according to TG quartiles.Table S2.Hazardratios for (A) MACEs, (B) CV events, and (C) CV mortality according to HDLquartiles (reversed). Table S3.Characteristics of study participants who were included in the analyses versusthose who were excluded from the analyses.Figure S1. Cubic spline curves of hazard ratios forMACEs, CV events, and CV mortality according to AIP levels. Figure S2. Subgroupanalyses for the risk of (A) MACEs, (B) CV events, and (C) CV mortality. [file 12933_2022_1522_MOESM1_ESM.docx]
